# Automated facial recognition for wildlife that lack unique markings: A deep learning approach for brown bears

**DOI:** 10.1002/ece3.6840

**Published:** 2020-11-06

**Authors:** Melanie Clapham, Ed Miller, Mary Nguyen, Chris T. Darimont

**Affiliations:** ^1^ BearID Project Sooke BC Canada; ^2^ Department of Geography University of Victoria Victoria BC Canada; ^3^ Raincoast Conservation Foundation Bella Bella BC Canada

**Keywords:** deep learning, face recognition, grizzly bear, individual ID, machine learning, wildlife monitoring

## Abstract

Emerging technologies support a new era of applied wildlife research, generating data on scales from individuals to populations. Computer vision methods can process large datasets generated through image‐based techniques by automating the detection and identification of species and individuals. With the exception of primates, however, there are no objective visual methods of individual identification for species that lack unique and consistent body markings. We apply deep learning approaches of facial recognition using object detection, landmark detection, a similarity comparison network, and an support vector machine‐based classifier to identify individuals in a representative species, the brown bear *Ursus arctos*. Our open‐source application, *BearID*, detects a bear’s face in an image, rotates and extracts the face, creates an “embedding” for the face, and uses the embedding to classify the individual. We trained and tested the application using labeled images of 132 known individuals collected from British Columbia, Canada, and Alaska, USA. Based on 4,674 images, with an 80/20% split for training and testing, respectively, we achieved a facial detection (ability to find a face) average precision of 0.98 and an individual classification (ability to identify the individual) accuracy of 83.9%. *BearID* and its annotated source code provide a replicable methodology for applying deep learning methods of facial recognition applicable to many other species that lack distinguishing markings. Further analyses of performance should focus on the influence of certain parameters on recognition accuracy, such as age and body size. Combining *BearID* with camera trapping could facilitate fine‐scale behavioral research such as individual spatiotemporal activity patterns, and a cost‐effective method of population monitoring through mark–recapture studies, with implications for species and landscape conservation and management. Applications to practical conservation include identifying problem individuals in human–wildlife conflicts, and evaluating the intrapopulation variation in efficacy of conservation strategies, such as wildlife crossings.

## Graphical abstract

One challenge of image‐based ecological studies is the inability to distinguish among individuals within species that lack unique markings. We describe an automated method to detect and identify individual brown bears (*Ursus arctos*), using facial recognition. Our application and detailed pipeline provide an important step in harnessing facial recognition techniques to address a broad spectrum of ecological questions that require individual ID in “unmarked” species.

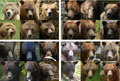

## 1 INTRODUCTION

Conservation Technology is an emerging field that aims to address large‐scale conservation challenges with innovative tools. With biodiversity conservation a global concern, computational approaches that enable wildlife monitoring at larger spatial scales, but with finer resolution, are recognized as a priority (Arts et al., 2015). Scaling‐up biodiversity monitoring (Steenweg et al., 2017), however, requires automation of data processing and analysis to increase reproducibility, while reducing time, cost, and labor (Weinstein, 2018).

Computer vision increasingly supports analyses of big data collected from image‐based ecological studies (Weinstein, 2015). One challenge is the inability to distinguish among individuals within species that lack unique markings (Rowcliffe et al., 2008). Addressing this gap and taking a similar approach to human individual identification, face recognition (in various forms) has been developed for nonhuman primates (great apes *Hominidae* spp. (Ernst & Küblbeck, 2011; Loos & Pfitzer, 2012; Freytag et al., 2016; Schofield et al., 2019), lemurs *Lemuroidea* spp. (Crouse et al., 2017), and macaques *Macaca mulatta* (Witham, 2018)). For species other than primates, one of the only references to facial recognition of unmarked species in the peer‐reviewed literature focuses on domestic dogs *Canis familiaris* (Moreira et al., 2017). Facial recognition approaches could prove useful to the suite of nonprimate wildlife species that lack distinctive body markings. Knowledge of unique individuals can facilitate the use of established techniques such as mark–recapture and thereby inform management.

Deep learning techniques automatically detect and extract learned features from data, and provide a powerful alternative to traditional methods of feature extraction (see Christin et al., 2019 and Schneider et al., 2019 for ecological applications). Face recognition using deep learning has recently achieved an accuracy of up to 92.5% for chimpanzees *Pan troglodytes* (Schofield et al., 2019) and 96.3% for giant pandas *Ailuropoda melanoleuca* (Chen et al., 2020); the latter possessing distinctive eye patch markings that could aid identification. A primary challenge, however, is that deep learning requires large labeled datasets for training and testing, which are difficult to acquire for wild populations, especially at the individual level (Schneider et al., 2019). Training on images of captive individuals offers a useful approach when such conditions exist for species of interest, but it is unclear how well these networks generalize to images taken of wild individuals in situ; controlled environments may provide inadequate training data for real‐world application (Wearn et al., 2019). Long‐term individual‐based ecological studies of wild populations (sensu Clutton‐Brock & Sheldon, 2010) provide an alternative and more common context that can support image databases collected over years. Moreover, variability contained within these images, such as fluctuations in body weight, may be more representative of the external morphology of wild animals.

Here, we describe our application *BearID*, which uses deep learning and facial images to detect and identify individual brown bears *Ursus arctos*, a species that lacks consistent, unique pelage markings. Brown bears provide an ideal candidate for expanding facial recognition beyond primates as they present opportunities and challenges likely spanning a wide variety of taxa: (a) They vary in morphology across their range (Hilderbrand et al., 1999), and (b) they experience extreme weight fluctuations between seasons and as they age and grow (Kingsley et al., 1983).

Using a programming pipeline of face detection and reorientation, face encoding, and face classification (Schroff et al., 2015), we trained and tested an object detection network, landmark detection network, similarity comparison network, and support vector machine (SVM)‐based classifier. We provide the methodological details for building the application, as well as our initial results and annotated source code. Although trained on a single species by design, *BearID* is transferable to other mammals and certain parts of the pipeline may be particularly transferable to other caniforms due to facial similarities. Given the number of species and the broad terrestrial and marine distribution of this important suborder of Carnivora, the frequency with which representative populations are studied, the expense of current identification methods (e.g., genetic tagging), and their relative ease of photographing, they comprise a well‐suited study system for this approach. *BearID* thereby provides an important step in applying deep learning methods of facial recognition to a variety of wild animals beyond primates that lack distinctive markings.

## 2 METHODS

### 2.1 Data collection

Images were collected in Knight Inlet, British Columbia, Canada (N 50°41′ W 125°44′) and Brooks River, Katmai National Park, Alaska, USA (N 58°33′ W 155°47′). Both sites have ongoing research and established bear‐viewing ecotourism. Images were collected for research purposes at Knight Inlet (by M Clapham and naturalists) and sourced *post hoc* from National Parks Service staff and seven independent photographers at Brooks River. Identifications of unique bears were provided by the collectors and represented unambiguously known individuals to each area, assessed by visual appearance and life history knowledge of individuals observed daily throughout seasons and years. All images were taken with DSLR cameras (various models and focal lengths), other than 19 images from Knight Inlet taken using Reconyx camera traps (PC85 Rapidfire Pro). These provided images of individuals not frequently observed (see Clapham et al., 2012). Resolution ranged from 0.3 to 24.1 megapixels. One image was in PNG and the rest JPEG format.

**Figure 1:**
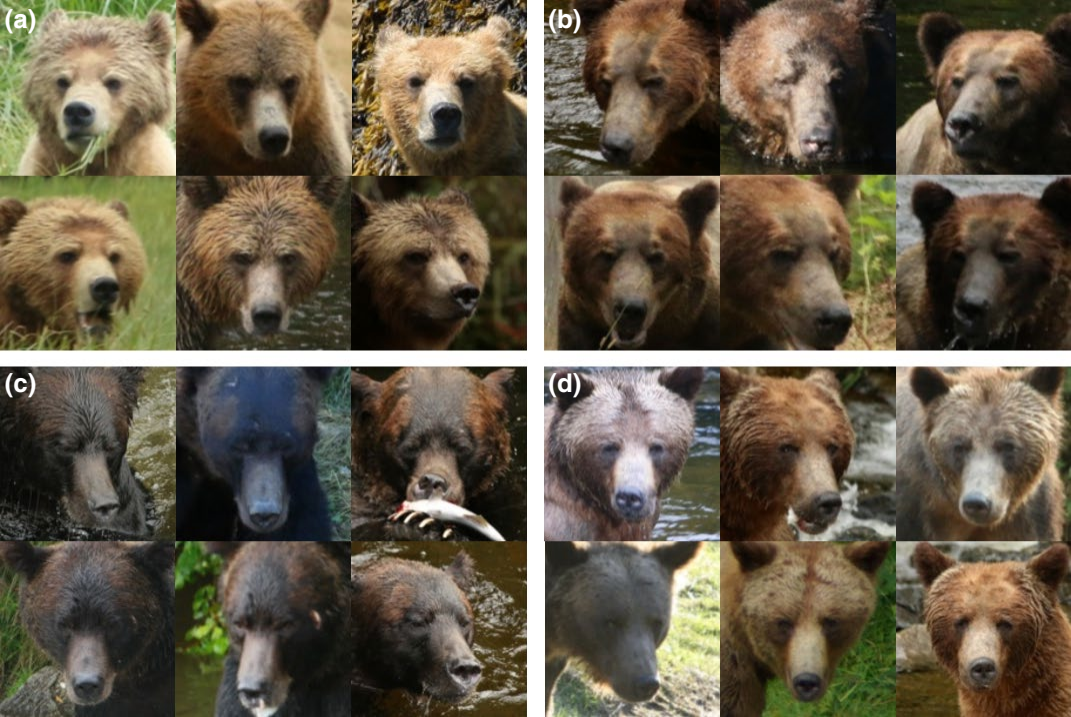
Variation among and within four individual brown bears (a–d). Images (face chips) display examples of the variation in pose, lighting, and photograph quality in the dataset. A is a subadult female, b and c are adult males, and d is an adult female. a, c, and d include images taken over successive years. Face chips were produced using *bearface* and *bearchip* applications

We collected 4,675 images of 132 individuals (median = 22 images/individual [range 1–242]) with visible faces (criteria: both eyes visible) taken May–October 2009–2017 (Table 1). Individuals were captured in successive years, across seasons within a year, and under varying light conditions (Figure 1).

**Table 1:** Summary of datasets collected and faces extracted

Dataset	Years	Number of face images (face chips)	Number of individuals (with face chips)
Knight Inlet, BC	2009–2017	1,297	59
Brooks River, AK	2014–2017	3,378	73
Total		4,675	132

### 2.2 Application pipeline

**Figure 2:**
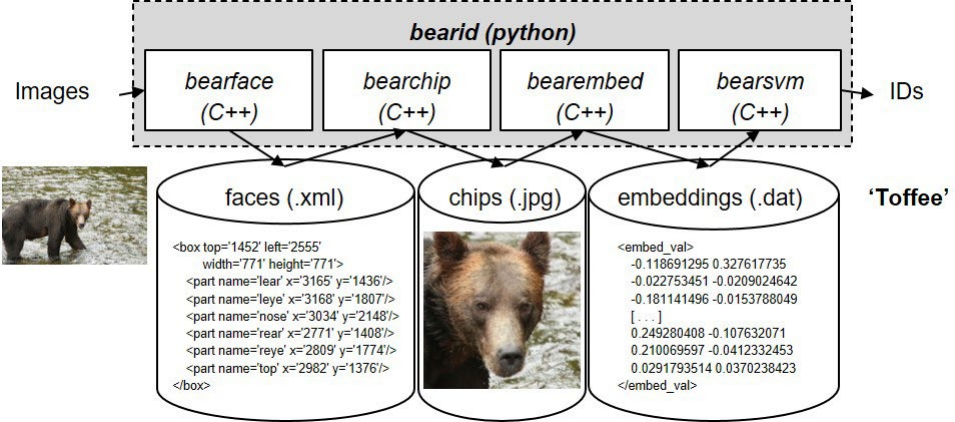
Schematic of *BearID* pipeline including programming languages and file formats (input image, detect face, crop and rotate face, create embedding for face, match embedding, classification of image, output individual ID “Toffee”)

Following the FaceNet approach (Schroff et al., 2015), our pipeline consisted of: (a) Face detection, (b) Face reorientation and cropping, (c) Face encoding (embedding), and (d) Face classification. We created separate C++ applications using the Dlib‐ml toolkit (King, 2009) for each step of the pipeline: (a) *bearface*, (b) *bearchip*, (c) *bearembed,* and (d) *bearsvm*. For end‐to‐end inferencing from photographs to identifications, we created a single Python script, *bearid* (Figure 2). We followed Dlib’s deep learning example programs to provide an outline for building *bearface*, *bearembed*, and *bearsvm* (see Data Accessibility).

To develop *BearID,* we custom‐built a computer system with a graphics processing unit capable of deep learning by providing parallel computing (see Appendix S1 for hardware details). We primarily used Python and C++ (below; Data Accessibility).

### 2.3 Training and test data

From 4,675 images, we created a fully labeled “golden dataset” that included a bounding box for each face, the locations of landmarks, and identification of each bear (see Appendix S2 for details). We randomly split the golden dataset into 3,740 (80%) images for training and 935 (20%) for testing using a Python script, *generate_partition.py*. One image had to be removed from the test split due to an incorrect label, for a total of 934 images in the test set. We used the data splits to train and test the various networks in our application (below).

For face detection, we scaled the resolution of all training and test images down to 2,000 × 1,500 pixels to avoid overloading our hardware. For the test set, if scaling caused a face to become too small (<200 × 200 pixels), we scaled until the face was 200 × 200 pixels and then cropped the overall image to 2,000 × 1,500 pixels (examples: Photographs S1 and S2).

### 2.4 Face detection

*Bearface* finds faces and landmarks (eyes, tip of the nose, ears, and top of the forehead) in images. It consists of two networks: an object detector (OD) and a shape predictor (SP). The OD uses a sliding window (Dalal & Triggs, 2005) and a convolutional neural network (CNN) trained with Dlib’s max‐margin object detection loss function (King, 2015). We selected this approach as Dlib’s example model trained on domestic dogs performed sufficiently to expedite labeling for the golden dataset (see Appendix S2). The CNN was trained using the bounding box labels in the golden dataset (see Appendix S3 for training procedure). The SP uses Dlib’s implementation of face alignment with an ensemble of regression trees (Kazemi & Sullivan, 2014) and was trained using the landmark labels in the golden dataset. The *bearface* application takes as input: an image file or list of images as an XML file and a network weights file. JPEG and PNG are both accepted as input file types, but raw or other format images would first need to be manually converted. It outputs an XML file with a list of images and corresponding face and landmark information.

### 2.5 Face reorientation and cropping

**Figure 3:**
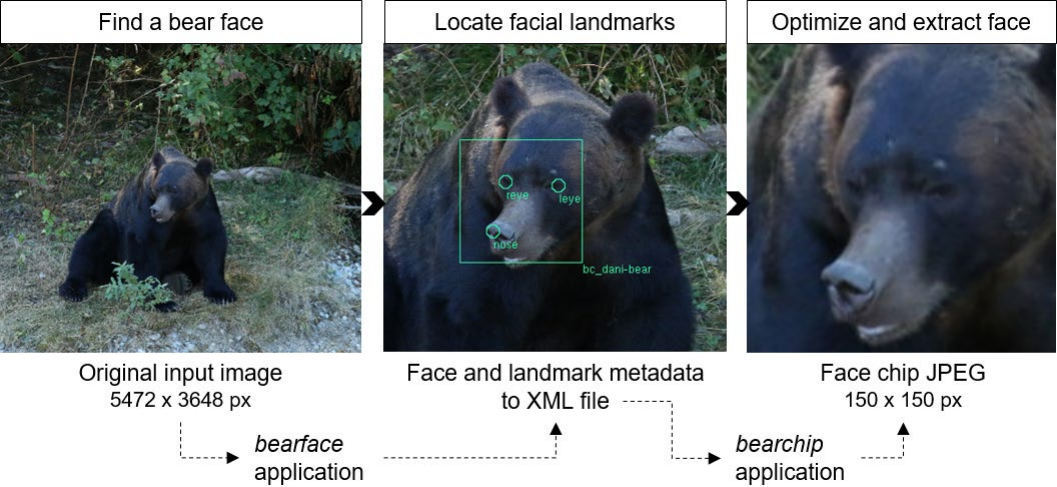
Automated face detection and extraction using *bearface* and *bearchip* applications. The resulting JPEG (face chip) provides input for the recognition networks forming the identification component of *BearID*. The middle image is cropped for figure clarity only

This stage uses the facial landmarks in the XML created by *bearface* to reorientate and extract the bear faces (or “chips”: Schroff et al., 2015). The application, *bearchip*, centers and rotates the face to optimal orientation. The current implementation uses only the eyes to align and center images (Table S1). It then scales and crops (150 × 150 pixels) the faces and writes each face chip as a JPEG file (Figure 3).

### 2.6 Face encoding

Face encoding forms the core process that facilitates facial recognition in the pipeline. It uses a similarity metric (Schneider et al., 2020) to learn a function that maps an input image (bear face chip) into a target space (Chopra et al., 2005). The *metric loss* function (Dlib toolkit: King, 2009) drives the similarity metric to be small for face chips of the same bear and large for face chips from different bears. The output is an embedding, which is a numeric vector representation of a facial image that can be compared to other embeddings to identify individuals using a face classifier (below).

For the implementation, *bearembed*, we trained a similarity comparison network using a deep CNN with a ResNet‐34 architecture (He et al., 2016), following the Dlib example “deep face recognition” implementation (King, 2009), to produce a 128‐dimensional Euclidean embedding per face chip image (Schroff et al., 2015). The *bearembed* application has three modes: training, testing, and embedding. To generate the face chip training data, we used *bearface* on the training portion of the golden dataset. For metric learning, we used pairwise hinge loss, rather than triplet loss (as in Schroff et al., 2015), implemented by the Dlib metric loss layer. We used hard negative mining to ensure a balanced ratio within mini‐batches of positive (same individual) and negative (different individuals) pairs from the training data (see Appendix S4 for training procedure). We augmented the training dataset by applying color perturbation and jittering each time a face chip was included in a mini‐batch (Appendix S4). *Bearembed* can be used with the trained network for testing or to generate embeddings for a set of chips.

### 2.7 Face classification

Face classification is the process of assigning an individual ID label to an embedding created by *bearembed,* from an existing dataset. We used a one‐versus‐one classifier using a linear SVM following the Dlib “multiclass classification” example to develop the application, *bearsvm*. We used the embeddings and labels from the chips in the training set to train the SVM. Once trained, *bearsvm* can be used to test the SVM or generate a classification of a bear face embedding (i.e., identify an individual).

### 2.8 Full application

The full *BearID* application was implemented as a python script, *bearid.py*. It takes a directory of images as the input. It executes *imglab* (image annotation tool: King, 2009) to create an XML file containing all the images in the directory, executes *bearface* to find bear faces, executes *bearchip* to create face chips, executes *bearembed* to create embeddings, and executes *bearsvm* to determine each bear identification. The output is an XML file with the original list of files, bounding boxes for the faces and the bear identification. The XML file can be read into *imglab* to review the annotations provided by *bearid.py*.

### 2.9 Testing methodology

We initially tested subapplications (*bearface*, *bearembed*, *bearsvm*) independently to gain an accurate representation of performance using the golden dataset. We then tested the full application from input image to ID classification to assess cumulative error on classification accuracy. We focus on subapplication results as our focal measures of performance.

#### 2.9.1 Face detection

The OD and SP were analyzed separately for the *bearface* application. We evaluated the OD using precision, recall, and interpolated average precision with an intersection over union at 0.5 of all predicted faces compared to those in the test split of the golden dataset (*n* = 934; Table 2). Precision can be considered as: if a face was detected, how often was it a face; recall can be considered as: how many faces were detected, out of all the faces present; and average precision as the area under the curve of the precision–recall curve, the latter comprising a key performance metric. We evaluated the SP by finding the distance between the predicted landmarks and those in the test split (Table 2). We normalized the distance for each landmark by scaling by the interocular distance. We report the mean normalized distances of all landmarks across all faces (Table 2).

**Table 2:** Testing results for the object detector and shape predictor that comprise *bearface*

Test method	Result
Precision (OD)	0.986
Recall (OD)	0.983
Average precision, IoU@0.5 (OD)	0.977
Mean normalized distance (SP)	0.111 ± 0.122

Note

Average precision calculated as area under the curve of the precision–recall curve with interpolated precision and an IoU (intersection over union) at 0.5. OD is object detector, and SP is shape predictor ± standard deviation.

#### 2.9.2 Face encoding

*Bearembed* was evaluated based on the “Labeled Faces in the Wild method” (Huang et al., 2007). We used k‐fold cross‐validation (*k* = 5) on the golden dataset, where each training fold contained 935 bear chips, and the test fold contained 934. Five different networks were trained using all combinations of 4 of 5‐folds (giving a training set of 935*4 = 3,740), then tested against the remaining fold for each network (934). For a fold, we created 3,000 unique matching pairs (same individual) and 3,000 unique nonmatching pairs (different individuals) from the 934 test chips for each fold using a python script, *generate_pairs.py*. The number of unique pairs (3,000) approximated the number of positive pair combinations, *n choose r* (*r* = 2), which can be created from the average number of chips per ID label (*n* = 8.12) multiplied by the number of ID labels (*m* = 115) in the test set. This can be written as:

$$\frac{n!}{r!(n‐r)!}\times m=\frac{8!}{2!(8‐2)!}\times 115=28\times 115=3,220$$

We ensured each fold had an even number of positive and negative examples, a given face image was never compared with itself, and all tested pairs were unique (positives and negatives). We estimated accuracy as:

(truepositiverate(TPR)×positiveratio)+(truenegativerate (TNR)×negativeratio)=(TPR×0.5)+(TNR×0.5).

We split the folds by the following: (1) face chip (randomly shuffling chips then splitting them evenly across the 5‐folds, resulting in a similar distribution of individuals and face chips across each fold and the same individuals appeared in both the training and test data [“closed set”: Deb et al., 2018]) and (2) ID label (randomly assigning each ID label to 1 of the 5‐folds [Huang et al., 2007], resulting in different individuals in each test set compared with its corresponding training set) to compare results. We tested by ID label to assess the performance of *bearembed* in creating embeddings for new individuals not previously used in training.

## 3 RESULTS

### 3.1 Face detection and reorientation

The OD attained an average precision of 0.977 (Table 2). The mean normalized distance between predicted and ground‐truthed facial landmarks was 0.111 ± 0.122 (SP; Table 2). Detection errors included misalignments, additional erroneous faces, and missed detections (examples: Photographs S3–S5). *Bearchip* is a mathematical operation and therefore cannot be evaluated (i.e., all detected faces were extracted). The parameters for *bearchip* (Methods) could be considered hyperparameters for *bearembed*.

### 3.2 Face encoding and classification

**Figure 4:**
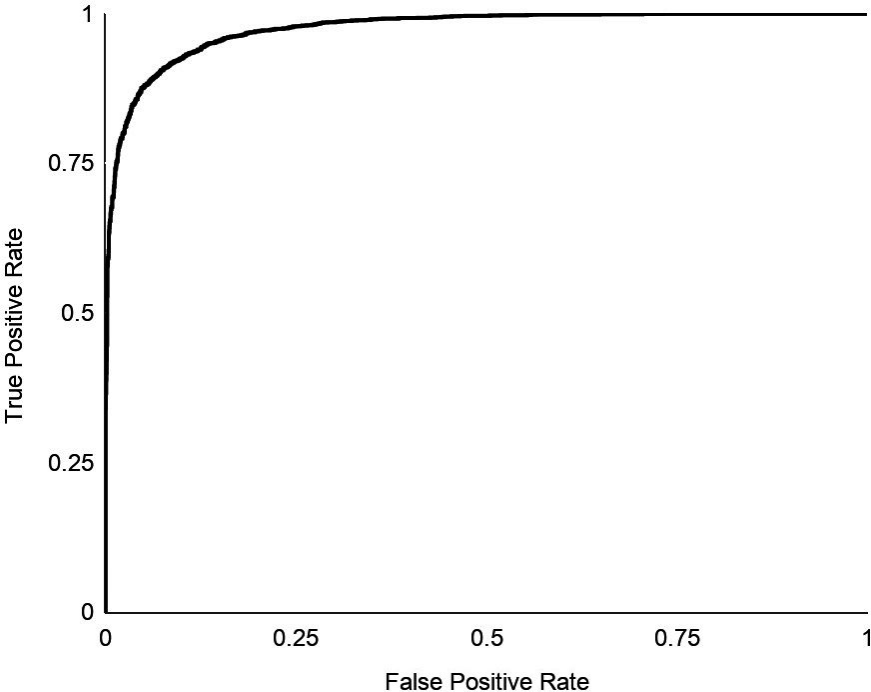
ROC curve displaying the probabilities of predicting matching or nonmatching pairs (the same individuals or different individuals) under different thresholds using the face encoder *bearembed*

**Figure 5:**
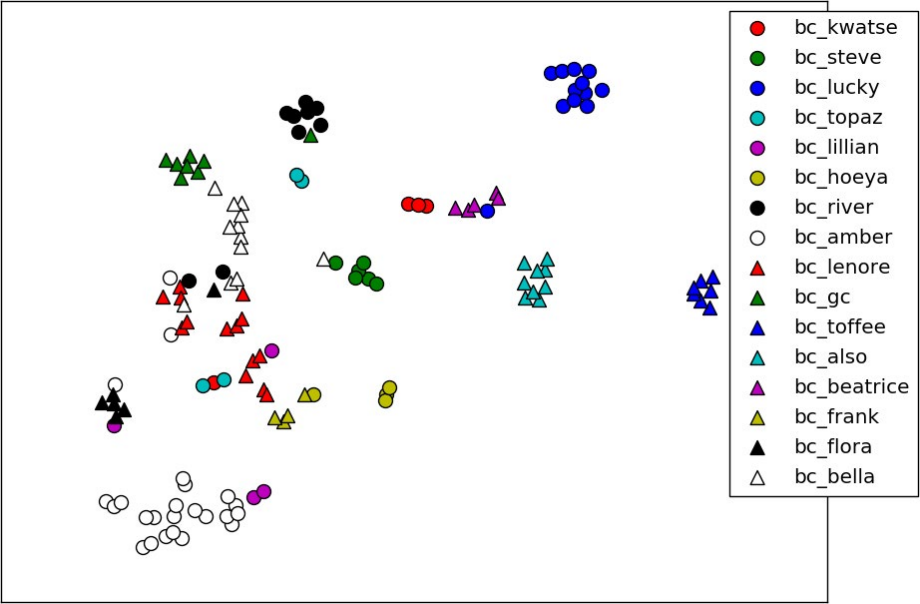
t‐SNE plot projecting 128D embeddings created using *bearembed* into 2D, for a subset of 16 individual bears. Each datapoint represents an embedding for a face chip; colors and shapes represent individuals. Perplexity = 5; learning rate = 10; iterations = 2,000; *n* = 125 embeddings (datapoints)

*Bearembed* had predictive utility when classifying between matched (same individual) and unmatched (different individuals) pairs (Figure 4). The error for the training data was nominal (Figure S1), which could indicate overfitting (the network learns to distinguish the specific training images rather than something more general). Higher accuracy occurred when splitting data by face chip, rather than ID label (Table 3). For visualization purposes, we created a subset of bears (*n* = 16: Knight Inlet) with > 3 images per bear. The resulting embeddings created by *bearembed* showed variation among and within individuals; images of some individuals were consistently clustered, whereas others were clustered with images of multiple individuals (Figure 5).

**Table 3:** Testing results for our face encoder *bearembed* using two methods of 5‐fold validation

Test method	Accuracy (%) ± *SD*
1. Face chip	84.2 ± 0.008
2. ID label	71.3 ± 0.024

Abbreviation: *SD*, standard deviation.

Using *bearsvm*, an ID prediction was made for each embedding in the golden dataset test split (934). Accuracy was determined by dividing the number of correct predictions (according to the ground‐truthed ID labels) by the total number of predictions. Two bears had single embeddings in the test set but none in the training set, so they could not be classified. Of the remaining 932 embeddings, *bearsvm* produced 782 correct predictions, yielding an accuracy of 83.9%. A confusion matrix was generated to further investigate classification performance by indicating which bears were confused when ID predictions were made among 16 individuals using *bearsvm* (Table 4). Classification performance varied in a similar way to embedding performance (Figure 5); some individuals were consistently identified accurately, whereas others were more likely to be confused with other bears (Table 4).

**Table 4:** Confusion matrix to assess classifier (*bearsvm*) performance using a subset of 16 individual brown bears. Numbers are image counts/individual (rows) and predicted identification (columns) using the classifier

		Predicted ID																
		Also	Amber	Beatrice	Bella	Flora	Frank	GC	Hoeya	Kwatse	Lenore	Lillian	Lucky	River	Steve	Toffee	Topaz	Correct classification (%)
Actual ID	Also	**7**	0	*1*	0	0	0	0	0	0	0	0	0	0	0	*1*	0	77.78
	Amber	0	**21**	0	0	*1*	0	0	0	0	0	0	0	0	0	0	0	95.45
	Beatrice	0	0	**5**	0	0	0	0	0	0	0	0	0	0	0	0	0	100.00
	Bella	0	0	0	**7**	0	0	0	0	0	*4*	0	0	0	0	0	0	63.64
	Flora	0	0	0	0	**5**	0	0	0	0	0	0	0	0	0	0	0	100.00
	Frank	0	0	0	0	0	**4**	0	0	0	0	0	0	0	0	0	0	100.00
	GC	0	0	0	0	0	0	**8**	0	0	0	0	0	0	0	0	0	100.00
	Hoeya	0	0	0	0	0	*1*	0	**3**	0	0	0	0	0	0	0	0	75.00
	Kwatse	0	0	0	0	0	0	0	0	**3**	0	0	0	0	0	0	*1*	75.00
	Lenore	0	0	0	0	0	*2*	0	0	0	**11**	0	0	0	0	0	0	84.62
	Lillian	0	*1*	0	0	0	*1*	0	0	0	0	**1**	0	0	0	0	0	33.33
	Lucky	0	0	*1*	0	0	0	0	0	0	0	0	**11**	0	0	0	0	91.67
	River	0	0	0	*2*	0	0	0	0	0	0	0	0	**6**	0	0	0	75.00
	Steve	0	0	0	0	0	0	0	0	0	0	0	0	0	**6**	0	0	100.00
	Toffee	0	0	0	0	0	0	0	0	0	0	0	0	0	0	**7**	0	100.00
	Topaz	0	0	0	0	0	0	0	0	0	0	0	0	0	0	0	**4**	100.00
														Mean correct				85.72

Counts in bold indicate correct predictions, counts in italics are incorrect predictions.

### 3.3 Initial testing of the full application

We evaluated the full application, *BearID,* to assess the cumulative effects of subapplication error on overall identification accuracy (i.e., the effect of errors/imprecision in facial detection on individual classification). We ran 934 test images through the *bearid* script to receive an ID classification for each image in which a face was detected (*n* = 929; Table 5). Two images of bears not represented in the training set were disregarded for the *bearsvm* and final *bearid* results. The overall accuracy for the full *BearID* pipeline was only slightly reduced (82.4%; Table 5) compared to when the classifier (*bearsvm*) was tested independently on the golden dataset (83.9%).

**Table 5:** Evaluating the impact of detection (*bearface*) and classification (*bearsvm*) error on full pipeline accuracy

Application	Task	Input	Results	Accuracy (%)
*Bearface*	Face and landmark detection	934 (source images)	929 (correct faces)^a^	99.5^b^
*Bearsvm*	ID classification	931 (embeddings)^a^	768 (correct ID)	82.5^c^
*Bearid* (full pipeline)	Face and landmark detection + ID classification	932 (source images)	768 (correct ID)	82.4

^a^ An extraerroneous face was detected in two files for a total of 931 faces.

^b^ Detection accuracy refers to the percentage of faces correctly detected (not how well the detection matches the ground truth; AP: Table 2).

^c^ Accuracy of classifications from source images varies compared to when *bearsvm* was tested on the golden dataset (above).

### 3.4 Transferability to other populations and species

The current version of *BearID* (20.05) can be used as an end‐to‐end application to collectively: (a) find a bear face and facial landmarks in an image; (b) extract and reorientate the face; (c) create a vector embedding for the face; and (d) use that embedding to identify the face from a database of known individuals. Subapplications can also be used as follows: *bearface* (face detector) can be used on brown bears and likely other bear species and other caniforms, due to morphological similarities, to detect faces and find facial landmarks in images. *Bearface* may facilitate facial detection in other species without species‐specific retraining, but has not yet been systematically tested; *bearchip* uses parameters optimized on brown bear faces and therefore may need adjustments for other species. Using data from *bearface* it produces a “face chip” needed by the face encoder; *bearembed* (face encoder) requires the "face chip" to create vector embeddings to test matching/nonmatching pairs of images. It can be used on brown bear individuals not currently in the dataset, but may have lower accuracy (see “by ID label” embedding results). It has not been tested on other species; *bearsvm* (classifier) compares embeddings to those already in a database to return a matching ID label and therefore can only be used for brown bears in the current dataset, requiring training on specific known individuals.

Although not designed primarily for this function, *BearID* can be used to conduct face verification (Deb et al., 2018) for “unknown” brown bears by running images through *bearface* and *bearchip*, using the embedding mode of *bearembed* to create embeddings for the images, and then the test mode of *bearembed* to test between the images. Results will indicate if the bears in the images are matching (same individual) or nonmatching (different individuals). Accuracy will be 71.3% [“ID label” accuracy] or possibly lower due to potential regional differences in morphology, as the network has only been trained on bears from two populations.

## 4 DISCUSSION

Our *BearID* pipeline provides a foundation for accurately applying deep learning methods of facial recognition beyond primates. Our trained face detector (ability to find a face) achieved an average precision of 97.7% without a strict criterion on facial pose, other than both eyes visible, which demonstrates the flexibility required for use on wild populations. Brust et al. (2017) estimated a similar average precision value (90.8%) for their detector of wild gorilla (*Gorilla gorilla*) faces. Testing our face detector on camera trap images would further enhance its use in ecological research and monitoring.

Our results suggest that our trained face encoder, *bearembed*, performed better when matching new images of known individuals, compared to matching images of new individuals. Testing *bearembed* revealed an accuracy of 84% when randomly selecting new images per known individual and 71% when assigning bears as training or testing individuals. We tested this latter mode to examine how our network would perform creating embeddings for bears not previously “seen,” which is relevant to ecological application (Schneider et al., 2020). Retraining a classifier to include an option to designate new individuals (sensu Deb et al., 2018) would further support the automated use of this software in wildlife research and monitoring. At present, new individuals must be added manually to the training dataset.

Our current classifier (*bearsvm*; ability to identify the individual) returned an identification accuracy of 83.9%. This result is within the range of values from other studies using CNNs for facial recognition of nonhuman primates (gorillas: 62.4% Brust et al., 2017; chimpanzees: 92.5% Schofield et al., 2019; lemurs: 93.8% Deb et al., 2018; and golden monkeys *Cercopithecus mitis kandti*: 90.4% Deb et al., 2018). Increasing the number of individuals in training datasets should increase accuracy (Brust et al., 2017) and resolve overfitting.

Images of the same individuals across years should result in a more robust identification network (Schofield et al., 2019), but could reduce accuracy (e.g., in humans: Rashmi et al., 2017). Further investigation of the influence of aging and weight gain on facial biometrics of species is needed for increased inference (insights into deep learning: Miao et al., 2019). These changes could also explain why some individuals were more consistently recognized than others. In addition, images of wild animals include variation in image quality due to distance from the focal animal, background, lighting, and pose. Using CNNs, Freytag et al. (2016) found lower accuracy for wild (77%) compared to captive chimpanzees (92%). Assessing the parameters that contribute to an “optimal facial image” and the impact of changes in facial appearance (e.g., facial trauma) could increase both the detection and recognition accuracy in future studies.

Whereas methods of pattern recognition applied to individual ID are well established (see Kühl & Burghardt, 2013), most mammals do not possess stable, unique markings. This inability to identify individuals objectively can restrict scientific inquiry and limit methods. *BearID* thus provides an important step in harnessing facial recognition techniques to address a broad spectrum of ecological questions that require individual ID, from fine‐scale behavior (e.g., individual activity patterns: Hertel et al., 2017) to landscape‐level population assessments (e.g., spatial mark–recapture using camera traps). We also see potential for this technology within conservation practice, such as to identify problem individuals in human–wildlife conflicts (see Swan et al., 2017) and to evaluate intrapopulation variation in efficacy of conservation strategies such as the use of wildlife crossing structures (e.g., Dexter et al., 2018), with implications for connectivity.

*BearID* may require additional species‐specific training for other taxa, but our pipeline provides an open‐source and replicable method as a foundation for discovery more broadly.

## CONFLICT OF INTEREST

The author(s) declare no competing interests.

## AUTHOR CONTRIBUTION

**Melanie Clapham:** Conceptualization (equal); Data curation (equal); Funding acquisition (lead); Investigation (equal); Methodology (supporting); Project administration (lead); Visualization (equal); Writing‐original draft (equal); Writing‐review & editing (lead). **Ed Miller:** Conceptualization (equal); Data curation (equal); Formal analysis (equal); Funding acquisition (supporting); Investigation (equal); Methodology (lead); Resources (lead); Software (lead); Validation (equal); Visualization (equal); Writing‐original draft (equal); Writing‐review & editing (supporting). **Mary Nguyen:** Conceptualization (equal); Data curation (equal); Formal analysis (equal); Investigation (equal); Methodology (lead); Resources (lead); Software (lead); Validation (equal); Visualization (equal); Writing‐original draft (equal); Writing‐review & editing (supporting). **Chris Darimont:** Conceptualization (supporting); Funding acquisition (lead); Supervision (lead); Validation (supporting); Writing‐original draft (supporting); Writing‐review & editing (equal).

## Supporting information

Appendix S1‐S4

## Data Availability

*BearID* is an open‐source application available on GitHub at https://github.com/hypraptive/bearid (version 20.05). The pretrained models are available at https://github.com/hypraptive/bearid‐models (version 20.05). The code and models are both also archived at https://doi.org/10.5281/zenodo.4014233. Owing to file sizes and copyright licenses, raw images will only be available upon request to the authors. The open‐source Dlib toolkit that we used to build applications can be downloaded at http://dlib.net/files/dlib‐19.7.tar.bz2. Dlib’s “max‐margin object detection” example program can be accessed at http://dlib.net/dnn_mmod_ex.cpp.html. Dlib’s “deep face recognition” example program can be accessed at http://dlib.net/dnn_face_recognition_ex.cpp.html. Dlib’s “multiclass classification” example program can be accessed at http://dlib.net/multiclass_classification_ex.cpp.html.
