# Supplementary material for: Automated facial recognition for wildlife that lack unique markings: A deep learning approach for brown bears
Source: Ecol Evol. 2020 Nov 6;10(23):12883–92. doi: 10.1002/ece3.6840 (PMC7713984; doi:10.1002/ece3.6840)
Supplement: Supplementary file 1 — Appendix S1‐S4 [file ECE3-10-12883-s001.pdf]

Automated facial recognition for wildlife that lack unique markings: a deep learning approach for brown bears

Melanie Clapham[1,2], Ed Miller[1], Mary Nguyen[1], and Chris T. Darimont[2,3]

[1] BearID Project, Sooke, Canada

[2] Department of Geography, University of Victoria, Canada

[3] Raincoast Conservation Foundation. Bella Bella, Canada

Supporting Information

This document contains:

- Additional information on the hardware used to develop *BearID* (Appendix S1)
- Procedures for establishing the golden dataset (Appendix S2)
- Training procedures for *bearface* (Appendix S3)
- Procedures for training the face encoder *bearembed* (Appendix S4)
- Locations of facial landmarks used to align and centre images (Table S1)
- A learning curve to examine the performance of *bearembed* (Figure S1)
- Scaling and cropping for face detection (Photos S1 and S2)
- Detection errors when using *bearface* (Photo S3-S5)
- References

Appendix S1. Hardware used to develop the *BearID* application

- CPU: Intel Core i5-6500 3.2GHz Quad-Core Processor
- Motherboard: MSI B150M BAZOOKA Micro ATX LGA1151
- Memory: Corsair Vengeance LPX 16GB (2 x 8GB) DDR4-2666
- Storage: Samsung 850 EVO-Series 500GB 2.5” Solid State Drive
- Video Card: EVGA GeForce GTX 1070 8GB SC GAMING ACX 3.0 Black Edition Video Card
- Case: Thermaltake Core V21 MicroATX Mini Tower Case
- Power Supply: EVGA SuperNOVA G2 550W 80+ Gold Certified Fully-Modular ATX Power Supply
- Keyboard: Logitech MK120 Wired Slim Keyboard w/Optical Mouse
- Monitor: Lenovo ThinkVision 24"

Further information on building our deep learning machine can be found at: hypraptive.github.io/2017/02/13/dl-computer-build.html

Appendix S2. Procedure for creating the labelled golden dataset

A labelled golden dataset (*n* = 4,675 images) was split into training (80%) and testing (20%) partitions used as the ground truth for training and testing the object detector, *bearface*. The labels included a bounding box for each face, the locations of landmarks, and identification of each bear. To construct the dataset, we:

1. Grouped all images by their identification
2. Used *imglab*, an image annotation tool from the Dlib-ml toolkit (King, 2009), to create XML files containing lists of images to process.
3. Ran the dataset through *bearface* using a pretrained network from a Dlib-ml example ('dog hipsterizer': github.com/davisking/dlib/blob/master/examples/dnn_mmod_dog_hipsterizer.cpp).
4. Manually edited the resulting XML using *imglab* to remove erroneous data, add missing data, and adjust facial landmark points.
5. For the test partition only: edited images containing more than one bear to simplify labelling. Editing involved removing other bears in the frame either by cropping or covering the face with a pasted section from the background of the image (example: Fig. 1).

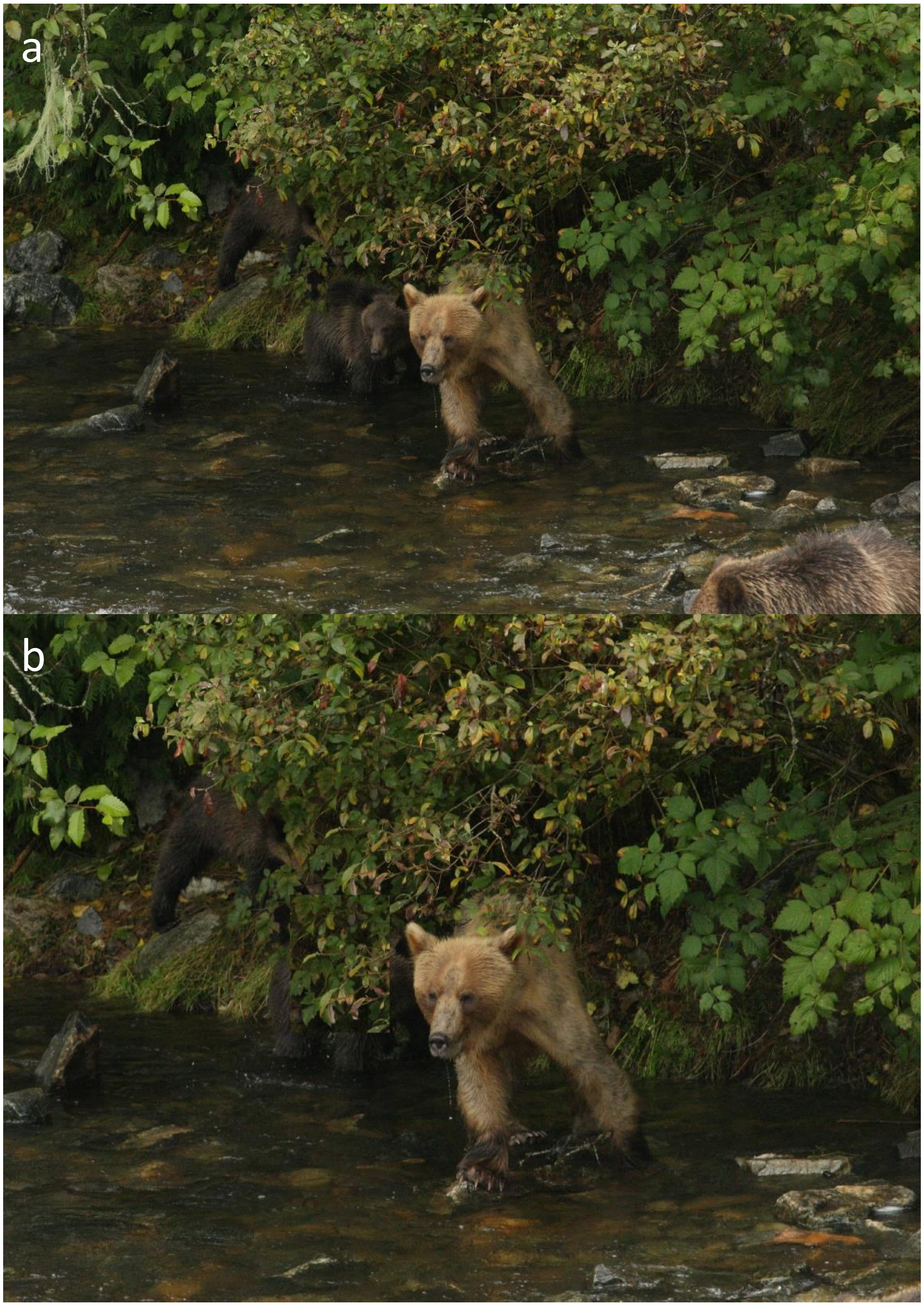

Figure 1. Example of manual editing of raw images that include more than one bear. A is the original image; B is the edited image. In the edited image, the resolution is scaled down to 2000x1500 pixels, the bear on the bottom-right is cropped out, and the cub face in the centre is replaced with background foliage (B).

Appendix S3. Training procedure for *bearface*

Based on dnn_mmod_ex.cpp from the Dlib-ml toolkit (King, 2009) using the training XML (n = 3740 images) and bounding box labels from the golden dataset, we trained as follows:

1. Images larger than 2000x1500 pixels were scaled down to 2000x1500 pixels.
2. We created mini-batches of data to better utilize the graphics processing unit (GPU) to speed up training. A mini-batch consisted of 75 image patches (300x300 pixels) that were randomly cropped from the training set images.
3. Stochastic gradient descent was applied for each mini-batch starting with a learning rate of 0.1, a weight decay of 0.1.
4. The learning rate was reduced when 8,000 batches were run with no improvement in the max-margin object detection loss. Training was stopped when the learning rate was less than 0.0001.
5. We ran 60,081 mini-batches.

Using the training partition XML (n = 3740 images), bounding box labels and landmarks, we trained the shape predictor based on the train_shape_predictor_ex.cpp from the Dlib-ml toolkit (King, 2009) with the following parameter changes:

Parameter	Value
Oversampling	300
Nu (regularization)	0.05
Tree Depth	5
Cascade Depth	20

Appendix S4. Training procedure for *bearembed*

Using the training partition XML (*n* = 3740 face chips), we trained as follows:

1. We created mini-batches of data to better utilize the graphics processing unit (GPU) to speed up training. A mini-batch consisted of a random selection of 5 face images for each of 5 individuals, for a total of 25 faces.
2. From these 25 faces, positive and negative pairs needed for metric learning were created.
3. Data were augmented by randomly perturbing the colour and jittering face chips for each mini-batch. Colour perturbation was achieved by applying a random transform function for colour balance and gamma correction on a pixel by pixel basis. Jittering was applied to 90% of face chips based on the following parameters:

Function	Definition
Translation	0-2% of height and width
Scaling	0-3% of height
Rotation	0-3 degrees
Horizontal flipping	50% of cases

4. Stochastic gradient descent was applied for each mini-batch with a learning rate of 0.001, a weight decay of 0.0001 and a momentum of 0.9.
5. The learning rate was reduced when 10,000 mini-batches were run with no improvement in the metric loss. Training was stopped when the learning rate was less than 0.0001.
6. We ran 76,000 mini-batches producing an augmented dataset of 1.9 million face chips.

Table S1. Locations of facial landmarks for optimally-oriented and centred bear face

Facial landmarks	As a percentage		In pixels (150x150)	
	X	Y	X	Y
Left Eye	62	48	93	72
Right Eye	38	48	57	72

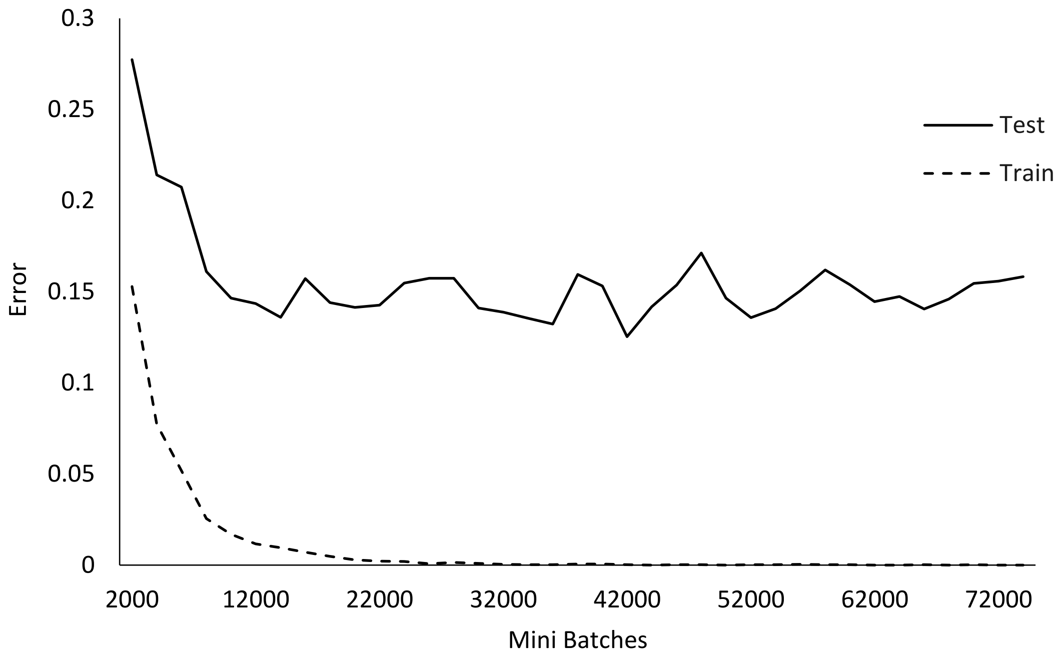

Figure S1. Learning curve to evaluate the performance of the deep convolutional neural network (CNN) used to train *bearembed*.

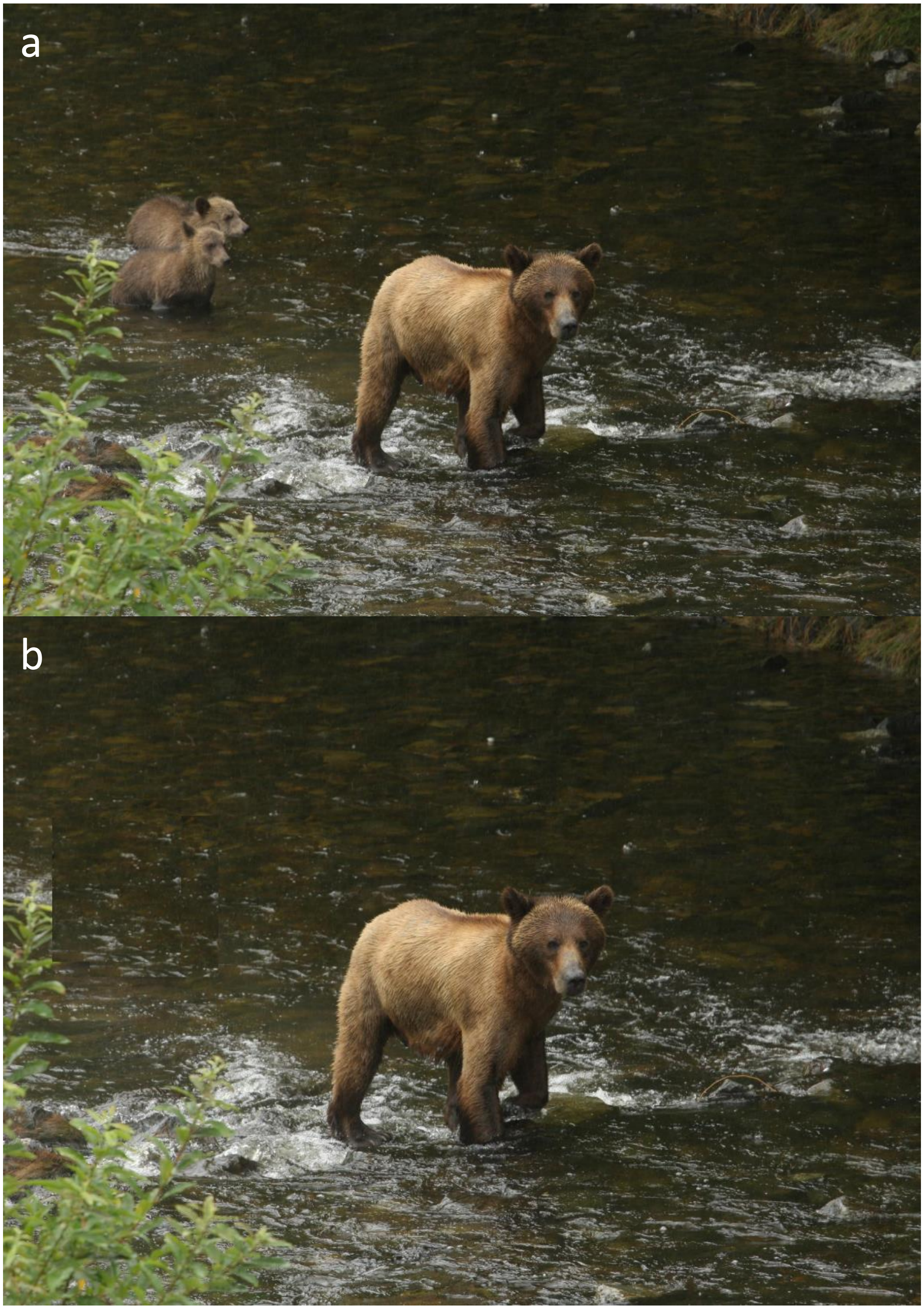

Photo S1. Example of scaling used to reduce the file size when testing *bearface*. A is the original image; B is the edited image, scaled to 2000x1500 pixels. Additional bears were also edited out using a pasted section of background (B; see Appendix S2).

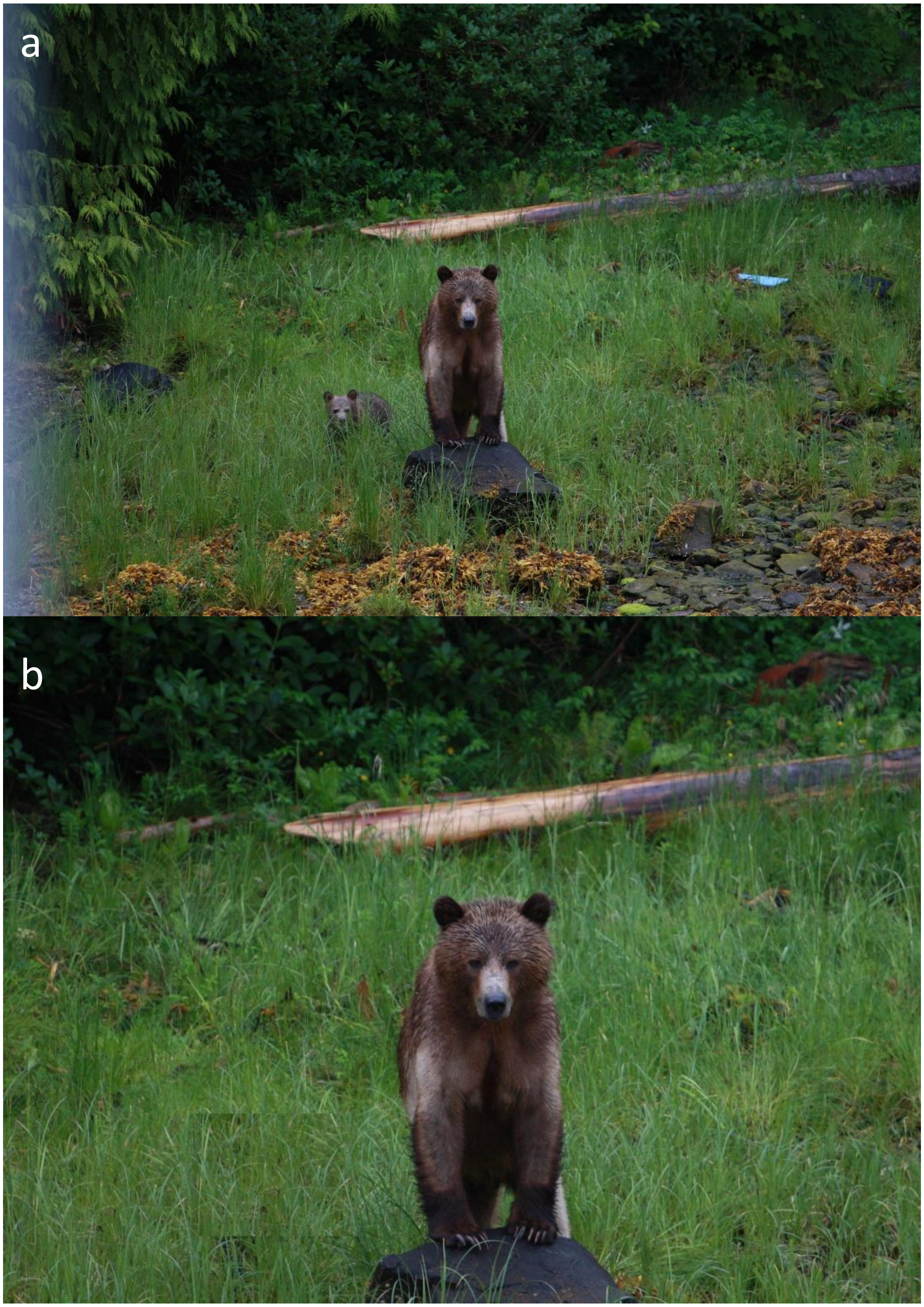

Photo S2. Example of scaling and cropping used to reduce the file size when testing *bearface*. A is the original image; B is the edited image. In B, the image was scaled until the face was 200x200 pixels and then the overall image cropped to 2000x1500 pixels. Additional bears were also edited out using a pasted section of background (B; see Appendix S2).

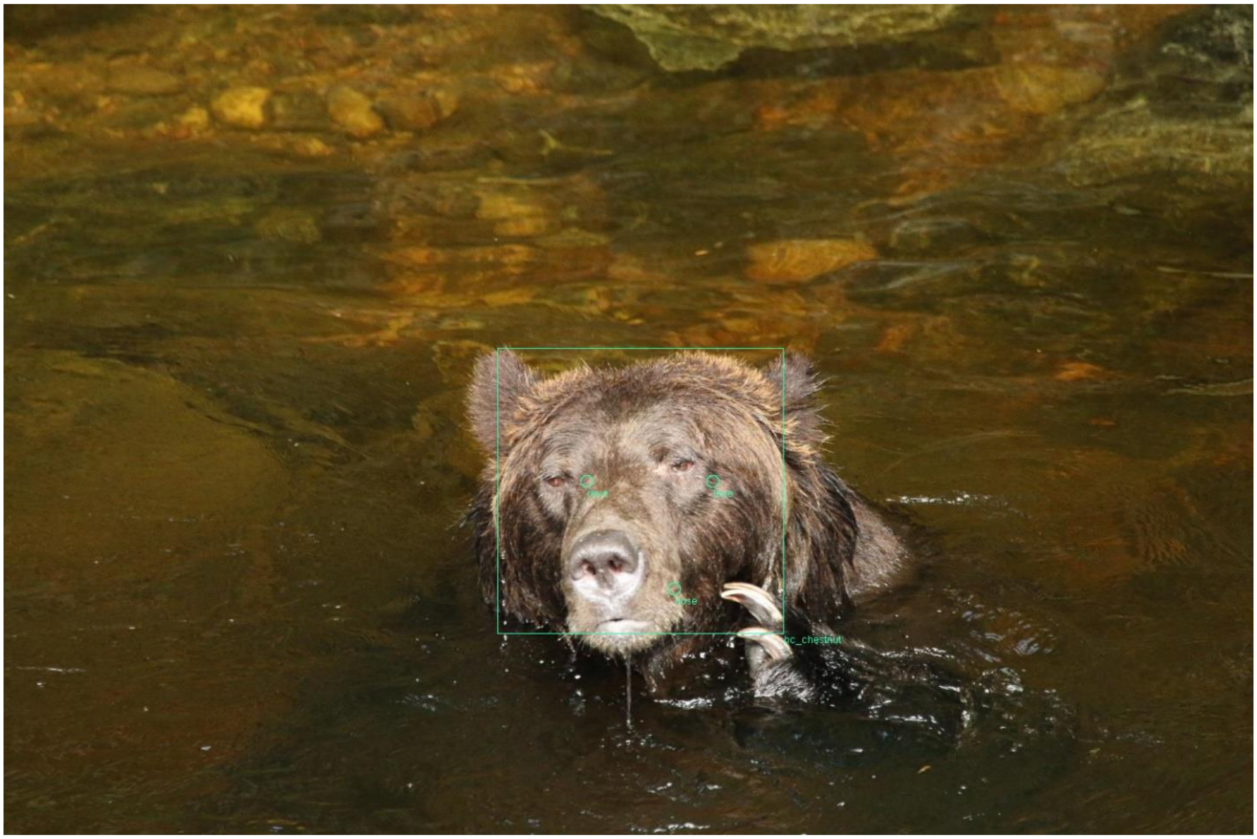

Photo S3. Example of an alignment error when using *bearface*. A bear face was detected, but the facial landmarks are incorrectly positioned.

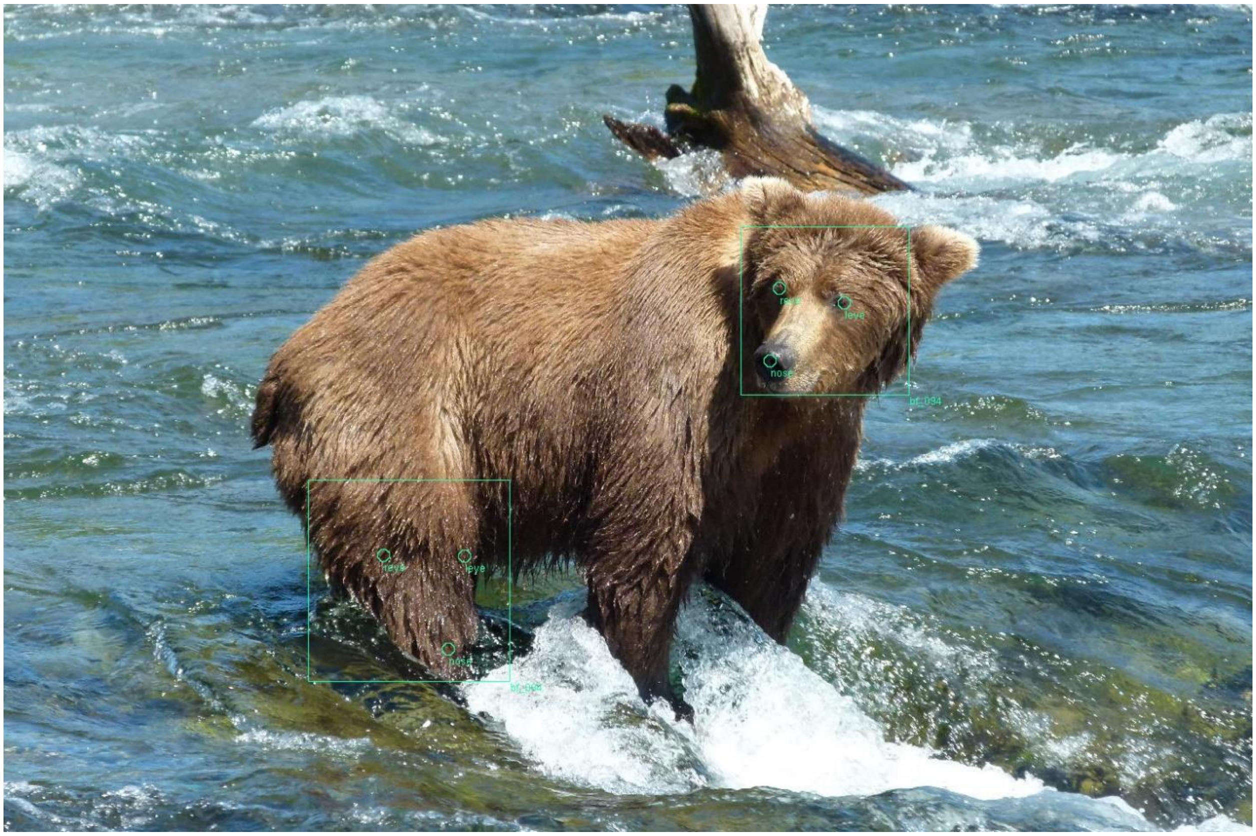

Photo S4. Example of an accurate face detection and a detection error when using *bearface*. There was an accurate face detection, but an additional erroneous face detected on the rear right leg of the bear. Photo: National Park Service, USA.

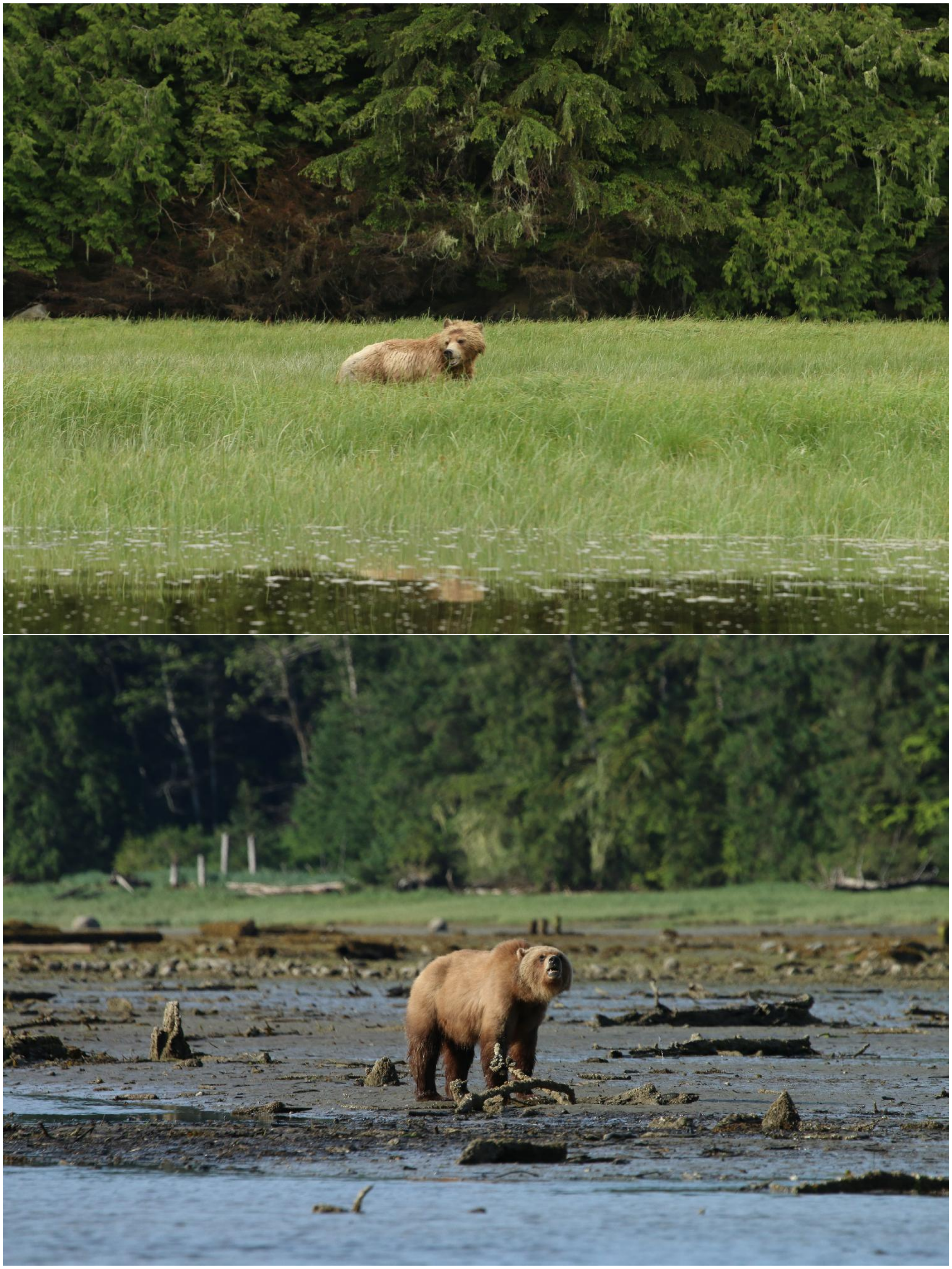

Photo S5. Examples of missed face detections when using *bearface*. A face is not detected in either image.

References

King, D. E. (2009). Dlib-ml: A machine learning toolkit. *Journal of Machine Learning Research, 10*, 1755–1758.
